# Mechanistic advances in the regulation of asthma by different exercise modalities: an integrative perspective on exercise load characteristics and asthma heterogeneity

**DOI:** 10.3389/fphys.2026.1947873

**Published:** 2026-09-18

**Authors:** Shihan Yang, Yingjie Zhou

**Affiliations:** 1 College of Sports, Minnan University of Science and Technology, Quanzhou, China; 2 Department of Pulmonary and Critical Care Medicine, Affiliated Hospital of Shandong University of Traditional Chinese Medicine, Jinan, China

**Keywords:** asthma, exercise load characteristics, exercise training, exercise-induced bronchoconstriction, mechanism-informed exercise selection

## Abstract

Asthma is a heterogeneous chronic airway disease in which exercise may act both as a trigger of respiratory symptoms and as a beneficial non-pharmacological intervention. Training responses vary across exercise modalities and asthma phenotypes; inconsistent findings for lung function, airway inflammation, bronchial hyperresponsiveness (BHR), and exercise-induced bronchoconstriction (EIB) highlight the need for a mechanism-oriented framework. This review synthesizes current evidence on moderate-intensity continuous aerobic training, high-intensity interval training, resistance training, combined aerobic-resistance training, breathing exercises, mind-body exercise, and aquatic exercise from the perspective of their dominant ventilatory, metabolic, mechanical-muscular, respiratory-behavioral, and neuropsychological loads. These load characteristics interact with different asthma phenotypes and clinical-mechanistic traits, including type 2-high eosinophilic asthma, obesity-related or non-type 2 asthma, EIB-dominant asthma, severe asthma with physical deconditioning, and dysfunctional breathing or anxiety-related symptoms. Four key mechanistic axes are examined: the ventilatory load-airway epithelium-EIB axis, immune inflammation-BHR axis, skeletal muscle-metabolic-systemic inflammation axis, and respiratory behavior-autonomic regulation-symptom perception axis. Structured exercise training provides the strongest and most consistent benefits for exercise capacity, asthma control, symptoms, and health-related quality of life.,whereas effects on airway inflammation, BHR, lung function, and EIB remain heterogeneous and appear to depend more on baseline phenotype, disease control, environmental exposure, and training protocol. The proposed mechanism-informed framework is intended to support individualized exercise selection, risk stratification, and outcome monitoring rather than serve as a fixed phenotype-specific prescription algorithm.

## 1 Introduction

Asthma is a heterogeneous chronic airway disease characterized by variable respiratory symptoms, airway inflammation, variable airflow limitation, and bronchial hyperresponsiveness (Global Initiative for Asthma, 2026). Although pharmacological treatment remains central to asthma management, many patients continue to experience reduced exercise tolerance, activity limitation, and impaired quality of life. Exercise occupies a complex position in asthma care: it may acutely provoke respiratory symptoms or EIB, particularly under conditions of high ventilation, cold or dry air exposure, environmental irritation, or suboptimal disease control (Parsons et al., 2013; Ameri et al., 2025) Conversely, structured exercise training is increasingly recognized as a useful non-pharmacological adjunct with potential benefits for asthma control, exercise capacity, dyspnea perception, and health-related quality of life (Hansen et al., 2020; Kuder et al., 2021; de Lima et al., 2023).

The available evidence indicates that exercise should not be treated as a uniform intervention. Aerobic training appears to improve asthma control and cardiorespiratory fitness, although effects on airway inflammation are inconsistent (Hansen et al., 2020). Breathing exercises may improve quality of life and hyperventilation-related symptoms, but their influence on inflammatory outcomes remains uncertain (Santino et al., 2020). HIIT may improve asthma control, asthma-related quality of life, and aerobic capacity in selected adults, while evidence regarding lung function and airway inflammation is limited or inconsistent (Regalado-Cabello et al., 2026). These differences are biologically plausible because exercise modalities impose distinct combinations of ventilatory, metabolic, mechanical-muscular, respiratory-behavioral, and neuropsychological loads.

Asthma is likewise heterogeneous. Patients differ in type 2 inflammatory status, EIB susceptibility, BHR, obesity-related metabolic dysfunction, physical deconditioning, dysfunctional breathing, and symptom perception. In this review, phenotype denotes a recognizable clinical or inflammatory pattern, whereas clinical-mechanistic trait denotes a potentially modifiable characteristic that may occur across phenotypes, such as EIB risk, deconditioning, obesity, or dysfunctional breathing. Therefore, a key question is not simply whether exercise is beneficial in asthma, but which modality, through which load characteristics, may influence which mechanism in which phenotype or trait context. Because direct comparative evidence across well-defined phenotypes and traits remains limited, the proposed framework should be interpreted as a hypothesis-generating, mechanism-oriented synthesis rather than a validated clinical algorithm. This review aims to synthesize current evidence on modality-specific exercise loads, asthma heterogeneity, and potential pathways to inform individualized exercise selection, progression, safety assessment, and monitoring.

## 2 Review methodology

This article was developed as an integrative, non-systematic review guided by principles for transparent literature synthesis (Snyder, 2019; Page et al., 2021). A targeted search of PubMed/MEDLINE, Web of Science, Scopus, and the Cochrane Library from database inception to 23 June 2026, supplemented by backward citation searching of key reviews and guidelines. Search concepts combined asthma with exercise training, aerobic training, high-intensity interval training, resistance training, pulmonary rehabilitation, breathing exercises, mind-body exercise, exercise-induced bronchoconstriction, airway inflammation, bronchial hyperresponsiveness, obesity, dysfunctional breathing, and phenotype or endotype. English-language peer-reviewed systematic reviews, meta-analyses, randomized or controlled clinical trials, clinical guidelines, and mechanistic or translational studies were considered when they directly informed exercise load characteristics, asthma heterogeneity, biological mechanisms, safety, or clinical prescription. Greater weight was given to systematic reviews, randomized trials, and current guidelines; mechanistic and narrative evidence was used to explain biological plausibility and was explicitly identified as inferential when direct clinical evidence was limited. The process was purposive rather than exhaustive, and no formal risk-of-bias assessment or meta-analysis was performed; consequently, the framework should not be interpreted as a systematic-review-derived clinical algorithm. PRISMA 2020 was consulted as a general transparency reference; this integrative review was not designed or reported as a PRISMA-compliant systematic review.

## 3 Exercise load characteristics across exercise modalities

Exercise-load characteristics refer to the dominant physiological or behavioral demands imposed by a modality, including ventilatory, metabolic, mechanical-muscular, respiratory-behavioral, and neuropsychological loads. These categories are not mutually exclusive, because most modalities combine several load components; the purpose of the classification is to identify the dominant stimulus most likely to shape benefit, symptom provocation, and monitoring needs.

### 3.1 Moderate-intensity continuous aerobic training

Moderate-intensity continuous aerobic training (MICT) is the most widely studied exercise modality in asthma and is commonly used as the reference approach in exercise-based interventions. It generally involves sustained rhythmic activities, such as walking, jogging, cycling, or treadmill exercise, performed at a relatively stable moderate intensity. Compared with interval or resistance-based exercise, MICT is characterized by continuous ventilatory and cardiometabolic loading, relatively steady oxygen consumption, and a dose structure that is straightforward to prescribe, monitor, and progress (Hansen et al., 2020; de Lima et al., 2023).

The main rationale for MICT is cardiorespiratory adaptation. Improved aerobic capacity may reduce the relative ventilatory demand required for submaximal activity, thereby alleviating exertional dyspnea and potentially reducing airway stress during daily physical activity. Systematic evidence suggests that exercise training improves cardiorespiratory and functional fitness in adults with asthma (Valkenborghs et al., 2022). A meta-analysis of randomized controlled trials further found improved asthma control and a small favorable effect on lung function after aerobic training, but no clear overall effect on airway inflammatory markers (Hansen et al., 2020).

Selected trials in patients with moderate-to-severe asthma have reported reductions in sputum eosinophils, fractional exhaled nitric oxide (FeNO), BHR, and systemic pro-inflammatory cytokines after aerobic training (Mendes et al., 2011; França-Pinto et al., 2015). However, these findings appear to depend on patient characteristics, baseline inflammatory status, medication stability, asthma severity, adherence, and training dose. Thus, MICT provides a well-supported model for improving fitness and patient-centered outcomes, whereas effects on EIB, BHR, and airway inflammation should be considered phenotype-dependent rather than universal (Eichenberger et al., 2013).

### 3.2 High-intensity interval training

High-intensity interval training (HIIT) consists of repeated short bouts of vigorous exercise separated by active or passive recovery periods. Compared with MICT, it produces intermittent peaks in ventilatory and metabolic demand within a shorter total training duration. This load pattern makes HIITa distinct exercise stimulus rather than simply a more intense version of continuous aerobic training (Aparecido da Silva et al., 2022; Regalado-Cabello et al., 2026). Evidence for HIIT in asthma is emerging but remains less extensive than that for MICT. Recent systematic review evidence indicates possible improvements in asthma control, asthma-related quality of life, and aerobic capacity, whereas effects on lung function and airway inflammation have not been consistently demonstrated (Toennesen et al., 2018; Aparecido da Silva et al., 2022; Regalado-Cabello et al., 2026). The current evidence base is therefore more supportive of functional and patient-reported benefits than of a specific airway anti-inflammatory effect. The heterogeneity of HIIT findings may reflect differences in interval duration, work-to-recovery ratio, total training dose, supervision, baseline fitness, medication optimization, and EIB susceptibility. Accordingly, comparisons with continuous training should distinguish time efficiency and adherence from biological superiority, because the current clinical evidence does not establish that HIIT produces greater airway anti-inflammatory effects than MICT (Toennesen et al., 2018; Aparecido da Silva et al., 2022; Regalado-Cabello et al., 2026).

Mechanistically, HIIT may create both acute airway-related risks and potential adaptive benefits. Vigorous intervals impose substantial ventilatory stress and may provoke EIB in susceptible or poorly controlled patients (Ameri et al., 2025). Conversely, when asthma is stable a carefully prescribed program that incorporates an adequate warm-up, gradual progression, and supervision when needed may improve aerobic capacity, exercise tolerance, and symptom perception (Toennesen et al., 2018; Aparecido da Silva et al., 2022; Regalado-Cabello et al., 2026). Therefore, HIIT should be discussed as a modality with a specific load profile and careful eligibility and monitoring requirements, rather than a universally applicable or inherently superior alternative to continuous aerobic training.

### 3.3 Resistance training and combined aerobic-resistance training

Resistance training primarily imposes mechanical-muscular and neuromuscular load, with less sustained ventilatory demand than continuous aerobic exercise or HIIT. It may therefore be useful for patients with peripheral muscle weakness, obesity, physical deconditioning, or activity limitation, particularly when prolonged hyperpnea is poorly tolerated (de Lima et al., 2023; Lo Bello et al., 2025; Costa et al., 2026). Its principal expected adaptations are increased peripheral muscle strength, improved functional reserve, and reduced relative effort during daily tasks rather than direct modification of airway inflammation.

Effect of combined strength and endurance training in adults with asthma: a randomized controlled trial In adults with moderate-to-severe asthma undergoing pulmonary rehabilitation, adding strength training to endurance training and education did not provide an additional improvement in asthma-related quality of life, although peripheral muscle strength improved only in the strength-training group (Lo Bello et al., 2025). A recent randomized trial in difficult-to-control asthma also reported that a resistance-training program improved peripheral muscle and functional outcomes; however, the study design included a photobiomodulation comparison and does not establish a direct airway anti-inflammatory effect of resistance exercise (Costa et al., 2026). Multicomponent programs combining aerobic exercise, muscle training, and stretching have also improved asthma control, but the independent contribution of resistance training cannot be isolated (Jaakkola et al., 2019).

Resistance training is therefore best viewed as a complementary strategy for muscle dysfunction, functional capacity, and perceived exertion rather than a primary airway-directed intervention. Combined aerobic-resistance training may address both cardiorespiratory fitness and peripheral muscle function, but current evidence does not support its universal superiority over aerobic training alone (de Lima et al., 2023; Lo Bello et al., 2025). Its effectiveness should be evaluated principally through muscular, functional, metabolic, and patient-centered outcomes.

### 3.4 Breathing exercises

Breathing exercises, including Buteyko breathing, the Papworth method, diaphragmatic breathing, and other breathing retraining approaches, primarily target respiratory-behavioral load rather than conventional cardiometabolic load. These interventions aim to modify breathing pattern, reduce excessive ventilation, improve breathing awareness, and support symptom self-management during daily activities or exertional breathlessness (Harper and Trayer, 2022; Hudson-Colby et al., 2024; Bal et al., 2025; Bondarenko et al., 2025).

Breathing retraining may be particularly relevant for patients with suspected or confirmed dysfunctional breathing or breathing pattern disorder, especially when dyspnea, chest tightness, or exercise limitation appears disproportionate to objective airflow limitation (Hudson-Colby et al., 2024; Bal et al., 2025). However, diagnostic criteria, intervention protocols, and outcome measures remain heterogeneous, and the certainty of evidence is limited (Mohan et al., 2024; Bondarenko et al., 2025). Therefore, breathing exercises should be viewed as adjunctive strategies targeting respiratory behavior and symptom perception, rather than as primary airway anti-inflammatory interventions.

### 3.5 Mind-body exercise

Mind-body exercises, such as yoga, Tai Chi, and Qigong, combine low-to-moderate physical activity with breathing regulation, relaxation, attentional control, and self-regulation. Their dominant load characteristics are respiratory-behavioral and neuropsychological rather than primarily cardiometabolic or mechanical-muscular (Yang et al., 2016; Heredia-Rizo et al., 2024).

Available evidence suggests that mind-body exercise may improve selected patient-centered outcomes, including symptoms, quality of life, exercise confidence, and anxiety-related symptom regulation (Yang et al., 2016; Heredia-Rizo et al., 2024). However, asthma-specific evidence remains less robust than that for aerobic training, and effects on lung function, medication use, airway inflammation, and BHR remain uncertain (Yang et al., 2016; Heredia-Rizo et al., 2024). These modalities may therefore be most relevant when anxiety-related dyspnea, dysfunctional breathing, symptom–lung function mismatch, or low confidence in physical activity contributes to disability.

### 3.6 Swimming and aquatic exercise

Swimming and aquatic exercise represent a specific form of aerobic training in which cardiopulmonary load is combined with a distinctive environmental exposure. From the perspective of exercise load characteristics, they imposes sustained cardiopulmonary demand and rhythmic breathing control, while the aquatic environment provides warm and humid inhaled air that may theoretically reduce cold- or dry-air airway irritation in selected susceptible patients (de Lima et al., 2023; Angelo Deus et al., 2024).

However, the clinical evidence for swimming or aquatic exercise in asthma remains limited. A recent systematic review and meta-analysis of randomized controlled trials concluded that improvements in lung function and quality of life are not supported by high-quality evidence, indicating that swimming should not be assumed to be superior to land-based aerobic exercise for asthma outcomes (Angelo Deus et al., 2024). It may nevertheless be an acceptable and enjoyable option when preference, adherence, or pediatric participation is important, provided that symptoms and environmental exposures are monitored.

Environmental context is particularly important. Although a recent systematic review found no consistent association between childhood swimming in chlorinated pools and subsequent asthma development (Silvestre et al., 2025), poorly ventilated pools and chlorine-related irritants may still aggravate symptoms in susceptible individuals. Pool ventilation, individual symptom response, and EIB risk should therefore be considered when aquatic exercise is prescribed (Price et al., 2025; Silvestre et al., 2025) (**Table 1**).

**Table 1:** Dominant load characteristics, adaptive targets, and evidence caveats of exercise modalities in asthma.

Exercise modality	Dominant load characteristics	Main adaptive targets	Potential advantages	Key limitations or precautions	References
Moderate-intensity continuous aerobic training	Sustained ventilatory and cardiometabolic load	Cardiorespiratory fitness, ventilatory efficiency, dyspnea perception, and asthma control	Most extensively studied; straightforward to prescribe, monitor, and progress	Effects on airway inflammation, BHR, and EIB are inconsistent and likely phenotype-dependent	(Hansen et al., 2020; Valkenborghs et al., 2022; de Lima et al., 2023; Urroz Guerrero et al., 2023)
High-intensity interval training	Intermittent high ventilatory and metabolic load	Aerobic capacity, exercise tolerance, and symptom confidence	Time-efficient, and potentially acceptable to selected stable adults	High acute ventilatory stress; requires stable disease, warm-up, and EIB monitoring	(Toennesen et al., 2018; Aparecido da Silva et al., 2022; Price and Simpson, 2023; Regalado-Cabello et al., 2026)
Resistance training	Mechanical-muscular and neuromuscular load	Peripheral muscle strength, functional reserve, and reduced relative effort	Useful for muscle weakness, deconditioning, obesity, or poor tolerance of prolonged hyperpnea	Limited asthma-specific evidence; benefits are mainly peripheral and functional rather than airway-directed	(de Lima et al., 2023; Lo Bello et al., 2025; Costa et al., 2026)
Combined aerobic–resistance training	Combined cardiopulmonary and muscular load	Cardiorespiratory fitness, muscle strength, functional status, and metabolic health	May address multiple limitations in obesity-related asthma, non-type 2 features, or physical deconditioning	The independent contribution of each component is difficult to isolate; not clearly superior to aerobic training alone	(Baffi et al., 2015; Jaakkola et al., 2019; de Lima et al., 2023; Lo Bello et al., 2025)
Breathing exercises	Respiratory-behavioral load	Breathing-pattern control, hyperventilation-related symptoms, and dyspnea perception	Relevant to dysfunctional breathing, hyperventilation, and symptom–lung function mismatch	Adjunctive intervention; effects on airway inflammation and BHR are limited or uncertain	(Santino et al., 2020; Hudson-Colby et al., 2024; Mohan et al., 2024; Bal et al., 2025)
Mind–body exercise	Respiratory-behavioral and neuropsychological load	Relaxation, self-regulation, symptom confidence, and anxiety-related dyspnea	May improve selected patient-centered outcomes and confidence in physical activity	Effects on lung function, medication use, and airway inflammation, and BHR remain uncertain	(Yang et al., 2016; Heredia-Rizo et al., 2024)
Swimming or aquatic exercise	Sustained aerobic load with warm-humid environmental exposure	Cardiorespiratory fitness, exercise participation, and environmental tolerance	Acceptable option when preference or adherence favors aquatic exercise	Superiority over land-based exercise is unproven; chlorine-related irritation and pool ventilation require consideration	(Angelo Deus et al., 2024; Price et al., 2025; Silvestre et al., 2025)

## 4 Asthma phenotypes and clinical-mechanistic traits as determinants of exercise response

### 4.1 Type 2-high or eosinophilic asthma

Type 2-high eosinophilic asthma is characterized by activation of type 2 immune pathways, eosinophilic airway inflammation, often accompanied by elevated FeNO, blood or sputum eosinophils, serum IgE, or allergic sensitization. Key cytokines include IL-4, IL-5, and IL-13. IL-4 and IL-13 contribute to IgE class switching, nitric oxide production, mucus-related changes, and structural cell responses, whereas IL-5 is closely linked to eosinophil biology. These mechanisms are associated with airway inflammation, mucus hypersecretion, and BHR (Tiotiu, 2018; Pelaia et al., 2022).

Moderate aerobic training is mechanistically relevant to this phenotype because it may improve fitness and may be associated with detectable changes in selected inflammatory outcomes in some patients. In patients with moderate-to-severe asthma under optimized medical treatment, aerobic training has been associated with reductions in sputum eosinophils and FeNO, with greater effects observed in those with higher baseline inflammatory markers (Mendes et al., 2011). A separate randomized trial reported reduced BHR and systemic pro-inflammatory cytokines together with improved quality of life (França-Pinto et al., 2015).

However, these findings should nevertheless be interpreted cautiously. A meta-analysis of randomized controlled trials found improved asthma control and a small favorable effect on lung function after aerobic training, but no clear overall effect on airway inflammatory markers (Hansen et al., 2020). Divergent results may reflect baseline eosinophilic activity, inhaled corticosteroid dose and stability, asthma severity, intervention duration, exercise intensity, adherence, biomarker sampling time, and the choice of FeNO, blood eosinophils, or sputum eosinophils as the inflammatory outcome. Type 2-high asthma should therefore be viewed as a phenotype in which inflammation-related responses may be more detectable, not one in which exercise can be assumed to consistently suppress type 2 inflammation. Future trials should prespecify inflammatory strata and combine FeNO, blood or sputum eosinophils, BHR, medication exposure, and clinical outcomes to distinguish airway effects from benefits mediated predominantly through fitness, dyspnea perception, or symptom control.

### 4.2 Non-type 2 and obesity-related asthma

Non-type 2 asthma and obesity-related asthma are overlapping but not identical phenotypes. Obesity-related asthma is often associated with worse symptom burden, poorer asthma control, altered lung mechanics, systemic low-grade inflammation, metabolic dysfunction, and changes in adipokine profiles. In some patients, obesity may amplify pre-existing allergic asthma, whereas in others it is associated with a later-onset, less eosinophilic phenotype. Therefore, the mechanisms driving symptoms and treatment response may extend beyond classical type 2 airway inflammation (Baffi et al., 2015; Bantulà et al., 2021).

In this context, exercise benefits may arise less from direct suppression of eosinophilic inflammation than from changes in body composition, cardiorespiratory fitness, insulin sensitivity, skeletal muscle function, and systemic inflammatory tone. In obese adults with asthma, adding exercise training to a weight-loss program improved asthma control, aerobic capacity, muscle strength, and health-related quality of life compared with weight-loss intervention alone (Freitas et al., 2017). By contrast, lifestyle interventions that achieve only modest changes in weight or physical activity have not consistently improved asthma control, emphasizing the importance of intervention dose, adherence, and the magnitude of achieved weight or fitness change (Ma et al., 2015).

Accordingly, resistance training and combined aerobic–resistance training may be particularly relevant when obesity, metabolic dysfunction, muscle weakness, or physical deconditioning contribute to symptom burden, whereas carefully prescribed HIIT may be considered in clinically stable patients when fitness improvement and adherence are realistic goals. These modalities should be viewed as strategies targeting extrapulmonary and systemic contributors to asthma morbidity rather than as primarily airway-directed interventions. Future studies should incorporate body composition, insulin resistance, adipokines, systemic inflammatory markers, aerobic capacity, muscle strength, and airway inflammatory indices to clarify whether clinical benefits are driven by metabolic adaptation, functional improvement, or changes in airway inflammation (Farhangi et al., 2025; Lo Bello et al., 2025).

### 4.3 Exercise-induced bronchoconstriction-dominant asthma

EIB refers to transient airway narrowing during or after exercise and is common in asthma, although it can also occur in individuals without established asthma (Parsons et al., 2013). In EIB-dominant disease, the critical stimulus is the ventilatory and environmental load imposed on susceptible airways. During vigorous or prolonged exercise, increased minute ventilation and promotes oronasal breathing, exposing the lower airways to large volumes of incompletely conditioned air, particularly under cold, dry, polluted, or irritant-rich conditions (Atchley and Smith, 2020; Anderson and Kippelen, 2023).

Airway surface water loss and increased airway osmolarity are central to the prevailing physiological model of EIB. These changes may produce epithelial stress and mediator release, leading to airway smooth-muscle contraction and the characteristic post-exercise decline in FEV1 (Anderson and Kippelen, 2023; Ameri et al., 2025). The detailed ventilatory load-airway epithelium pathway is discussed in next section; clinically, the important implication is that EIB risk varies with exercise intensity and duration, breathing route, air temperature and humidity, environmental exposure, and baseline asthma control. EIB-dominant asthma requires careful management of acute airway stress rather than avoidance of physical activity. Gradual progression, an individualized warm-up, interval-based initiation, environmental modification, and attention to breathing pattern may reduce symptom provocation and improve exercise tolerance. Systematic review evidence suggests that high-intensity interval or variable-intensity warm-up can attenuate EIB in some individuals, possibly through a refractory period, although responses vary and pharmacological management remains necessary when indicated (Stickland et al., 2012). Objective assessment of the post-exercise fall in FEV1 should be used when feasible because symptoms alone do not reliably distinguish EIB from deconditioning, dysfunctional breathing, or anxiety-related dyspnea.

### 4.4 Severe asthma with physical deconditioning

Severe asthma is frequently accompanied by persistent symptom burden, multimorbidity, reduced physical activity, fatigue, and impaired functional capacity despite optimized pharmacological treatment. In this phenotype, exercise intolerance may not be explained solely by airflow limitation, but also cardiorespiratory deconditioning, peripheral muscle weakness, dyspnea-related fear, and reduced confidence in physical activity. Therefore, exercise training should be framed as part of a pulmonary rehabilitation strategy rather than as an isolated exercise prescription (McLoughlin et al., 2022; Guarnieri et al., 2025).

Recent evidence for rehabilitation-oriented interventions in severe asthma is promising but limited. A systematic review and meta-analysis reported improvements in daily step count, asthma control, and health-related quality of life, but included only a small number of short-term randomized trials (McLoughlin et al., 2022). In patients receiving triple-inhaler therapy, 12-week high-intensity aerobic training improved peak oxygen uptake, work rate, dyspnea, and fatigue at peak exercise, without significant changes in spirometry or respiratory muscle strength (Jao et al., 2024). These findings suggest that exercise-related benefits in severe asthma may be expressed mainly through improved functional capacity and symptom tolerance rather than through measurable changes in resting lung function.

Relevant targets in severe asthma with physical deconditioning include cardiorespiratory fitness, peripheral muscle strength, ventilatory efficiency, dyspnea, fatigue, anxiety, and health-related quality of life. For carefully selected patients, multicomponent pulmonary rehabilitation may combine aerobic and resistance training with education, self-management, breathing retraining, or inspiratory muscle training. Larger phenotype-stratified trials are needed to define optimal candidates, dose, supervision, safety monitoring, maintenance strategies, and long-term effectiveness (Urroz Guerrero et al., 2023; Guarnieri et al., 2025).

### 4.5 Dysfunctional breathing and anxiety-related asthma symptoms

Some patients with asthma report breathlessness, chest tightness, hyperventilation, or exercise intolerance that appears disproportionate to objective airflow limitation. In these patients, dysfunctional breathing or breathing pattern disorder may coexist with asthma and contribute to symptom burden, reduced quality of life, and difficulty achieving perceived disease control. This phenotype is better understood as a symptom-perception and respiratory-behavior phenotype trait rather than as a distinct inflammatory endotype (Hudson-Colby et al., 2024; Bal et al., 2025).

Breathing exercises and mind–body interventions may be particularly relevant because they target breathing pattern, ventilatory control, relaxation, and self-regulation. Breathing pattern disorders can negatively affect multiple domains in difficult-to-treat asthma, but diagnostic criteria and objective assessment methods remain inconsistent (Hudson-Colby et al., 2024; Bal et al., 2025). Although breathing retraining is frequently studied in adults with dysfunctional breathing, the certainty of evidence remains limited and intervention protocols vary substantially (Bondarenko et al., 2025).

Potential pathways include reduced excessive ventilation, improved breathing pattern awareness, less anxiety-related symptom amplification, and greater confidence during physical activity. These interventions should not be interpreted as primary anti-inflammatory therapies. Their main role is likely to improve symptom interpretation, breathing control, and behavioral responses to exertional breathlessness. They may be used as adjuncts to optimized asthma management when dysfunctional breathing, anxiety-related dyspnea, or symptom-lung function mismatch is present (Harper and Trayer, 2022; Bondarenko et al., 2025).

## 5 Mechanistic pathways

The four proposed axes should not be interpreted as equally established mechanisms. The ventilatory load-EIB axis is underpinned by relatively direct human physiological and challenge-test evidence, whereas exercise-related immune modulation and skeletal muscle–metabolic signaling are supported by a mixture of clinical trials, biomarker studies, translational observations, and biological plausibility. The respiratory behavior-symptom perception axis mainly explains patient-centered outcomes and symptom-lung function mismatch rather than direct airway anti-inflammatory effects. Throughout this section, causal language is therefore reserved for well-supported acute physiological processes, while longer-term mechanistic links are described as potential, associated, or hypothesis-generating (**Figure 1**).

**Figure 1:**
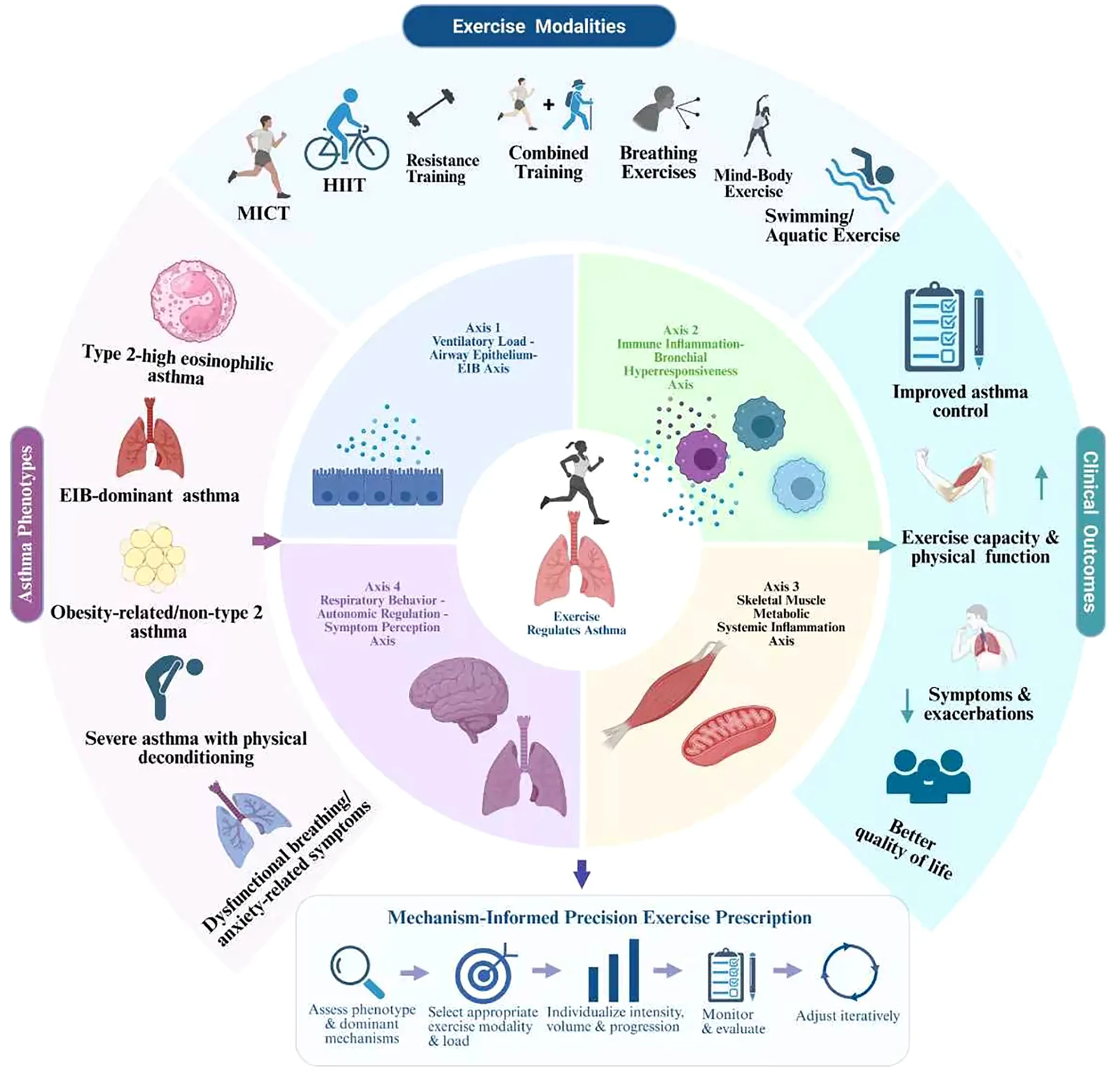
Integrative mechanistic framework of exercise modality-specific regulation of asthma.

### 5.1 Ventilatory load-airway epithelium-exercise-induced bronchoconstriction axis

The ventilatory load–airway epithelium axis helps explain why exercise can act as both a trigger and a therapeutic stimulus. During vigorous exercise, increased minute ventilation and a shift from nasal to oronasal breathing expose the lower airways to larger volumes of insufficiently conditioned air. This promotes airway surface water loss, increases airway-surface-liquid osmolarity and may induce epithelial stress, initiating inflammatory and neuroimmune responses related to EIB (Anderson and Kippelen, 2023; Ameri et al., 2025).Increased airway osmolarity is associated with activation of resident airway cells, including mast cells, and release of bronchoconstrictive mediators such as histamine, cysteinyl leukotrienes, and prostaglandins. These mediators contribute to airway smooth muscle contraction and post-exercise airway narrowing, particularly under conditions of high ventilatory demand or exposure to cold, dry, polluted, or irritant-rich air (Anderson and Kippelen, 2023; Ameri et al., 2025). This acute mechanism is clinically distinct from the longer-term adaptations produced by repeated exercise training.

Structured and progressive exercise training may improve cardiorespiratory fitness and reduce the relative ventilatory requirement for a given submaximal workload, thereby lessening exertional dyspnea and airway stress during daily activity. However, current evidence stronger for improvements in exercise capacity, symptoms, asthma control, and quality of life than direct restoration of epithelial integrity or uniform prevention of EIB. Future studies should integrate ventilatory measurements, environmental monitoring, post-exercise FEV1responses, airway mediator profiles, and patient-reported outcomes to clarify the relationship between acute airway stress and chronic adaptation (de Lima et al., 2023; Price and Simpson, 2023).

### 5.2 Immune inflammation-bronchial hyperresponsiveness axis

Exercise training may influence airway and systemic inflammation, but the magnitude and direction of these effects are heterogeneous. In type 2-high asthma, candidate outcomes include eosinophilic airway inflammation, FeNO, and type 2 cytokine activity. In non-type 2 or obesity-related asthma, relevant pathways may include systemic cytokines, oxidative stress, metabolic inflammation, and low-grade inflammatory tone rather than direct suppression of eosinophilic inflammation (Moraes-Ferreira et al., 2022; de Lima et al., 2023).

Aerobic exercise may reduce selected inflammatory markers in some patients, particularly when baseline inflammation is elevated. Recent studies have reported reductions in airway inflammatory or remodeling-related mediators after physical training, and acute moderate exercise has been associated with a short-term reduction in eosinophilic airway inflammation (Moraes-Ferreira et al., 2022; Scott et al., 2022). However, improvements in asthma control, quality of life, or exercise capacity are not always accompanied by consistent changes in FeNO, sputum eosinophils, or other inflammatory biomarkers (de Lima et al., 2023; Valkenborghs et al., 2024).

BHR is a useful integrative outcome because it reflects interactions among airway inflammation, epithelial function, airway smooth-muscle reactivity, and neural regulation. Exercise training may improve BHR in selected patients, but this response cannot be assumed across phenotypes. Mechanism-oriented studies should combine inflammatory biomarkers, bronchial challenge testing, exercise load monitoring, and patient-reported outcomes to distinguish airway modulation from benefits mediated primarily by fitness, dyspnea perception, or symptom management (de Lima et al., 2023; Price et al., 2025).

### 5.3 Skeletal muscle-metabolic-systemic inflammation axis

Exercise is a systemic intervention, and some benefits in asthma may be mediated through extrapulmonary pathways. Skeletal muscle functions as a metabolic and endocrine organ; improvements in muscle strength, mitochondrial function, glucose metabolism, and myokine signaling may influence systemic inflammatory tone and functional capacity, especially in patients with obesity-related asthma or physical deconditioning (Balakrishnan and Thurmond, 2022; Lu et al., 2024). This axis is particularly relevant to resistance training, combined aerobic-resistance training, and selected HIIT protocols, which may improve peripheral muscle function, exercise tolerance, and perceived exertion even when changes in resting lung function or airway inflammation are limited. Recent rehabilitation studies support improvements in peripheral muscle strength and selected functional or quality-of-life outcomes in severe or difficult-to-control asthma (Guarnieri et al., 2025; Lo Bello et al., 2025).

In obesity-related asthma, exercise may indirectly influence disease outcomes by improving body composition, insulin sensitivity, skeletal muscle function, and adipose tissue inflammation. However, direct evidence linking exercise-induced myokine or metabolic changes to specific airway outcomes remains limited. Therefore, this axis should be regarded as an emerging explanatory framework rather than a fully established mechanism in asthma (Škrgat et al., 2024; Russjan, 2024).

### 5.4 Respiratory behavior-autonomic regulation-symptom perception axis

Breathing pattern, ventilatory control, autonomic regulation, and symptom perception can substantially influence asthma-related disability. In some patients, breathlessness or exercise intolerance may be disproportionate to airflow limitation, suggesting contributions from dysfunctional breathing, hyperventilation, anxiety, or altered symptom interpretation (Hudson-Colby et al., 2024; Bal et al., 2025). Breathing exercises and mind-body interventions may improve outcomes through pathways that are partly independent of airway inflammation, including better breathing-pattern awareness, reduced excessive ventilation, relaxation, and improved management of dyspnea-related fear (Bal et al., 2025; Bondarenko et al., 2025). Because protocols and outcome measures remain heterogeneous, these approaches are best considered adjunctive strategies for patients with dysfunctional breathing, anxiety-related dyspnea, or symptom-lung function mismatch, rather than substitutes for optimized anti-inflammatory asthma treatment (Heredia-Rizo et al., 2024; Mohan et al., 2024; Bondarenko et al., 2025) (**Table 2**).

**Table 2:** Exercise modality selection according to dominant asthma phenotype or clinical-mechanistic trait.

Dominant phenotype or clinical-mechanistic trait	Potentially suitable exercise strategy	Dominant load characteristic	Principal target or rationale	References
Type 2-high or eosinophilic asthma	Moderate-intensity aerobic training during stable disease control	Sustained cardiopulmonary load	Improve fitness and evaluate whether inflammatory or BHR-related outcomes change in patients with elevated baseline markers	(Mendes et al., 2011; França-Pinto et al., 2015; Moraes-Ferreira et al., 2022; Scott et al., 2022; de Lima et al., 2023)
EIB-dominant asthma	Progressive aerobic training, structured warm-up, interval-based initiation, and environmental control	Ventilatory and airway osmotic load	Reduce acute airway stress and support safe participation; objectively assess post-exercise airway narrowing	(Parsons et al., 2013; Anderson and Kippelen, 2023; Price and Simpson, 2023; Ameri et al., 2025; Goldin and Bruner, 2025; Price et al., 2025)
Obesity-related or non-type 2 asthma	Combined aerobic–resistance training, selected HIIT in stable patients	Metabolic and mechanical- muscular load	Target metabolic dysfunction, body composition, muscle weakness, and other extrapulmonary contributors	(Ma et al., 2015; Freitas et al., 2017; Sánchez-Ortega et al., 2023; Škrgat et al., 2024; Russjan, 2024; Farhangi et al., 2025)
Severe asthma with physical deconditioning	Supervised pulmonary rehabilitation incorporating aerobic and resistance training	Low-to-moderate progressive multicomponent load	Improve exercise capacity, peripheral muscle function, dyspnea tolerance, fatigue, and quality of life	(McLoughlin et al., 2022; Jao et al., 2024; Guarnieri et al., 2025; Lo Bello et al., 2025)
Dysfunctional breathing or anxiety-related symptoms	Breathing retraining and selected mind-body exercise as adjuncts	Respiratory-behavioral and neuropsychological load	Improve breathing control, symptom interpretation, self-regulation, and confidence during activity	(Heredia-Rizo et al., 2024; Hudson-Colby et al., 2024; Bondarenko et al., 2025)

## 6 Mechanism-informed exercise prescription

Mechanism-informed exercise prescription should be interpreted as a hypothesis-generating decision-support framework embedded within optimized asthma management and, for complex or severe disease, pulmonary rehabilitation (Spruit et al., 2013; Price et al., 2025; Global Initiative for Asthma, 2026). Current evidence can inform risk stratification, modality selection, progression, and outcome monitoring, but it is insufficient to define rigid prescriptions for each asthma phenotype or clinical-mechanistic trait. Exercise should be deferred and medical reassessed considered during an acute exacerbation, marked symptom instability, unexplained exertional desaturation, chest pain, syncope, or severe bronchospasm. Relative precautions include poorly controlled asthma, frequent reliever use, recent exacerbation, high EIB risk, major cardiometabolic comorbidity, and exposure to cold, dry, polluted, or chlorine-irritant environments. Within these boundaries, modality, intensity, volume, and supervision should be individualized according to disease control, dominant treatable traits, exercise load characteristics, comorbidities, patient preference, and functional goals (Agusti et al., 2016; McDonald et al., 2020; Price et al., 2025; Global Initiative for Asthma, 2026).

In practice, prescription should begin by identifying the dominant phenotype and modifiable clinical-mechanistic trait. EIB-dominant disease may require a structured warm-up, gradual progression, environmental control, and objective post-exercise assessment. Patients with type 2-high or eosinophilic features may undertake moderate aerobic training when disease control and medication are stable, with inflammatory outcomes monitored where feasible. Those with obesity-related or non-type 2 asthma, muscle weakness, or deconditioning may benefit from combined aerobic–resistance training. HIIT should be considered only in clinically stable individuals after assessment of EIB risk, comorbidities, and exercise tolerance. Low aerobic capacity should be treated as a cross-cutting deconditioning trait rather than a separate asthma phenotype. Breathing retraining or mind-body exercise may be added when dysfunctional breathing or anxiety-related dyspnea contributes to limitation (Moraes-Ferreira et al., 2022; Anderson and Kippelen, 2023; de Lima et al., 2023; Škrgat et al., 2024; Hudson-Colby et al., 2024).

Monitoring should be aligned with the dominant trait and the intended mechanism of benefit. In addition to asthma control, symptoms, exacerbations, and health-related quality of life, selected measures such as FeNO, blood or sputum eosinophils, post-exercise fall in FEV_1_, BHR, peak oxygen uptake, six-minute walk distance, muscle strength, body composition, dyspnea scores, anxiety measures, and breathing pattern assessment may be used (McLoughlin et al., 2022; Moraes-Ferreira et al., 2022; de Lima et al., 2023). These outcomes can help distinguish improvement driven by airway modulation from cardiorespiratory adaptation, metabolic change, or altered symptom perception. (**Table 3**; **Figure 2**)

**Table 3:** Mechanism-informed considerations for exercise prescription in asthma.

Dominant clinical-mechanistic trait	Expected adaptations or/prescription aim	Exercise emphasis	Practical precautions and relative contraindications	Suggested monitoring outcomes	Evidence caveat	References
Type 2-high or eosinophilic inflammation	Improve asthma control and fitness, evaluate whether airway inflammatory outcomes change	Moderate-intensity continuous aerobic training	Prescribe during stable disease control; avoid abrupt escalation; reassess after a recent exacerbation or frequent reliever use	ACQ/ACT, FeNO, blood or sputum eosinophils, BHR, and exacerbation history	Inflammatory effects may be detectable in selected patients but are not consistent across trials	(Moraes-Ferreira et al., 2022; de Lima et al., 2023; Price et al., 2025; Global Initiative for Asthma, 2026)
EIB-dominant trait	Reduce acute ventilatory and osmotic airway stress and maintain participation	Progressive aerobic training, structured warm-up, and interval-based initiation	Avoid cold, dry, polluted, or irritant-rich environments; include adequate warm-up; defer unsupervised vigorous exercise when EIB is uncontrolled	Post-exercise fall in FEV1, EIB symptoms, reliever use, environmental exposure, and exercise tolerance	Evidence is stronger for acute EIB mitigation than for long-term elimination of EIB susceptibility	(Anderson and Kippelen, 2023; de Lima et al., 2023; Ameri et al., 2025; Price et al., 2025; Global Initiative for Asthma, 2026)
Obesity-related or non-type 2 trait	Improve metabolic health, body composition, muscle function, and functional capacity	Combined aerobic–resistance training; selected HIIT when stable	Screen cardiometabolic and musculoskeletal comorbidities; progress gradually; do not assume direct eosinophilic effects	Body composition, waist circumference, metabolic markers, CRP, muscle strength, 6MWD, and ACQ/ACT	Systemic and metabolic mechanisms are plausible, but direct links to airway outcomes remain incompletely established	(de Lima et al., 2023; Škrgat et al., 2024; Russjan, 2024; Price et al., 2025)
Severe asthma with physical deconditioning	Improve functional capacity, dyspnea tolerance, and quality of life	Pulmonary rehabilitation-based aerobic and resistance training	Prefer supervised pulmonary rehabilitation; defer training during exacerbation or marked instability; account for comorbidities, fatigue, desaturation, and fall risk	6MWD, VO_2peak_, dyspnea scale, fatigue, muscle strength, AQLQ, asthma control	Functional benefits are supported, but optimal dose and long-term maintenance remain uncertain	(McLoughlin et al., 2022; de Lima et al., 2023; Urroz Guerrero et al., 2023; Guarnieri et al., 2025; Lo Bello et al., 2025)
Dysfunctional breathing or anxiety-related dyspnea	Improve breathing control, symptom interpretation, and self-regulation	Breathing retraining, diaphragmatic breathing, yoga, Tai Chi, or Qigong	Use as adjunctive therapy after excluding uncontrolled asthma or EIB; do not interpret symptom improvement as proof of reduced airway inflammation	Breathing-pattern assessment, Nijmegen Questionnaire where appropriate, dyspnea, anxiety, and quality of life	Evidence mainly concerns respiratory behavior and patient-reported outcomes direct anti-inflammatory evidence is limited	(Heredia-Rizo et al., 2024; Hudson-Colby et al., 2024; Bondarenko et al., 2025; Price et al., 2025)
Low aerobic capacity or deconditioning	Improve cardiorespiratory fitness and exercise tolerance	MICT as the reference approach; selected HIIT only when clinically stable	Assess disease control, EIB risk, cardiovascular contraindications, and warm-up response before vigorous intervals	Peak oxygen uptake, work rate, 6MWD, fatigue, post-exercise FEV1 fall, and symptom tolerance	HIIT may improve fitness and asthma-related outcomes in selected stable adults, but it should not be considered universally superior to MICT	(de Lima et al., 2023; Price et al., 2025; Regalado-Cabello et al., 2026)

**Figure 2:**
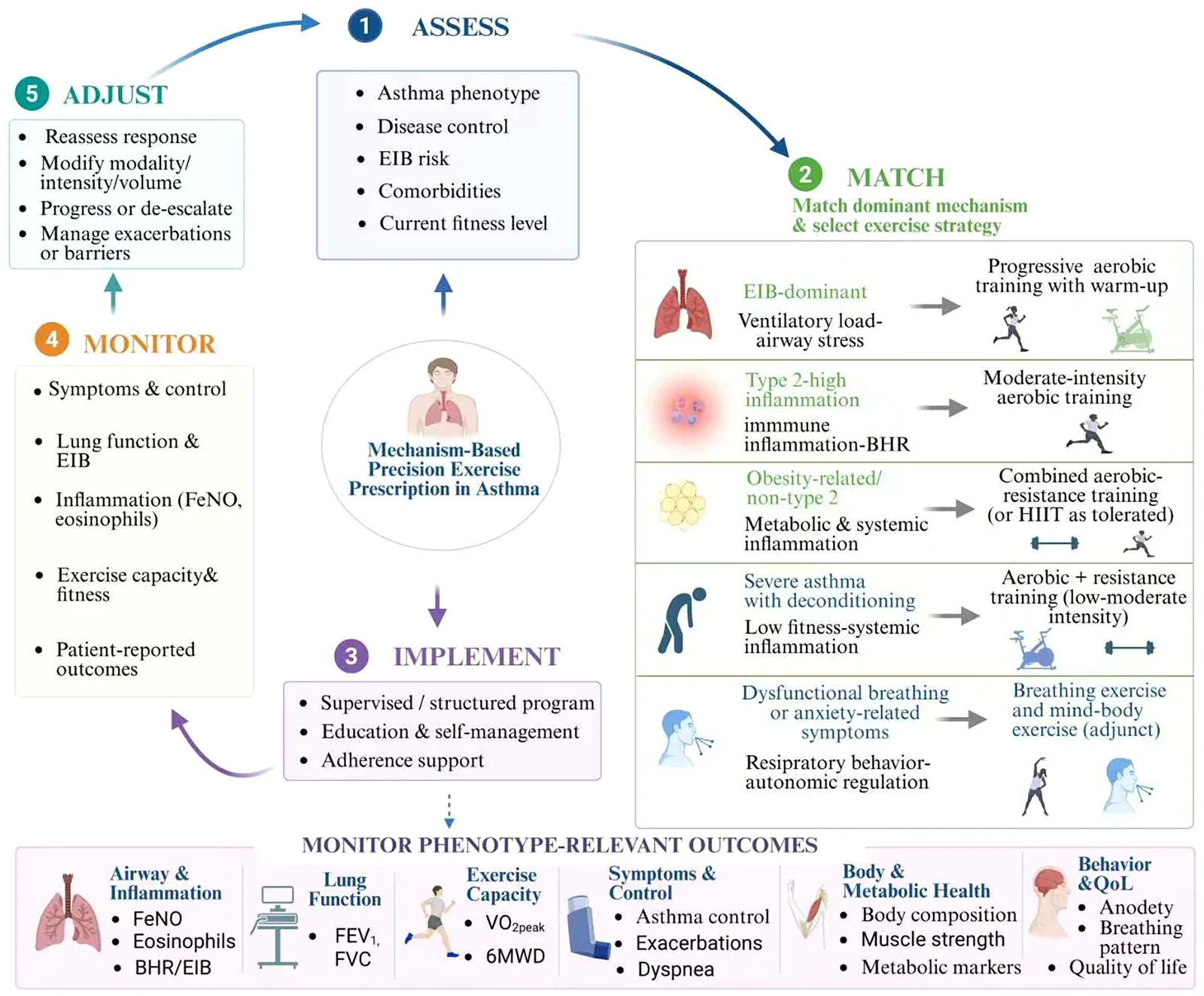
Mechanism-informed precision exercise prescription workflow in asthma.

## 7 Limitations and future directions

The evidence base is limited by substantial methodological heterogeneity and by the small number of studies designed to test whether mechanistic biomarkers mediate clinical improvement. Exercise protocols vary in modality, intensity, duration, frequency, progression, interval structure, supervision, and adherence assessment. Ventilatory demand, environmental conditions, warm-up strategy, and inspired-air exposure are often incompletely reported, making it difficult to separate modality-specific effects from total dose, improved fitness, behavioral engagement, or reduced symptom avoidance (de Lima et al., 2023; Price et al., 2025). Asthma heterogeneity is also insufficiently addressed. Many studies include mixed populations without adequate stratification by type 2 inflammation, obesity-related or non-type 2 asthma, EIB, severe asthma with deconditioning, or dysfunctional breathing. This limitation may partly explain inconsistent findings for airway inflammation, BHR, lung function, and symptom-related outcomes (McLoughlin et al., 2022; de Lima et al., 2023; Ameri et al., 2025; Bondarenko et al., 2025; Regalado-Cabello et al., 2026). Direct comparisons of modalities under matched energy expenditure or ventilatory load remain limited.

Future trials should adopt standardized, mechanism-oriented designs and report FITT-VP components together with asthma-specific variables such as ventilatory demand, environmental exposure, EIB risk, medication stability, supervision, and adherence. Studies should compare modalities under controlled energy expenditure or ventilatory load, stratify participants by phenotype and treatable traits, and distinguish acute airway responses from chronic training adaptation (Anderson and Kippelen, 2023; Ameri et al., 2025).

Mechanistic assessment should combine clinical outcomes with FeNO, eosinophils, cytokines, airway mediator profiles, bronchial challenge testing, cardiopulmonary exercise testing, muscle function, metabolic markers, autonomic indices, and breathing-pattern assessment. Longitudinal studies should test whether biomarker changes mediate clinical benefit rather than merely accompany improved fitness. Digital health platforms, wearable sensors, home-based or remote pulmonary rehabilitation, and artificial-intelligence-assisted decision support may improve monitoring and access, but require validation for safety, equity, data quality, and clinical effectiveness before routine implementation. Multi-omics approaches, including metabolomics, transcriptomics, epigenetics, microbiome profiling, and systems biology integration, may help identify novel exercise-responsive pathways but should currently be regarded as exploratory tools within well-phenotyped prospective studies (Agusti et al., 2016; McDonald et al., 2020; Liu et al., 2025; Idrees et al., 2026).

## 8 Conclusion

Exercise is a heterogeneous intervention in asthma, and its effects are shaped by modality-specific load characteristics, disease control, environmental exposure, and the patient’s dominant phenotype and clinical-mechanistic traits. MICT has the most consistent evidence for improving asthma control, exercise capacity, and quality of life. HIIT, resistance training, and combined programs may provide additional functional or metabolic benefits in selected, clinically stable patients, whereas breathing and mind-body interventions are most relevant to dysfunctional breathing, anxiety-related symptoms, and symptom-lung function mismatch. Effects on airway inflammation, BHR, lung function, and EIB remain inconsistent and should not be generalized across phenotypes. A mechanism-informed framework can support individualized modality selection, risk management, progression, and outcome monitoring, but it should remain flexible and subordinate to optimized pharmacological care. Future research should prioritize standardized protocols, trait-based stratification, and multimodal mechanistic assessment to determine who responds, through which pathway, and under which exercise-load conditions.
